# A neuromechanical model characterizing the motor planning and posture control in the voluntary lean in Parkinson’s disease

**DOI:** 10.1186/s12984-024-01321-0

**Published:** 2024-02-15

**Authors:** Niromand Jasimi Zindashti, Zahra Rahmati, Abolfazl Mohebbi, Saeed Behzadipour

**Affiliations:** 1 Mechanical Engineering Department, Sharif University of Technology, Tehran, Iran; 2 Djawad Movafaghian Research Center in Rehab Technologies, Sharif University of Technology, Tehran, Iran; 3 Department of Mechanical Engineering, École Polytechnique de Montréal, Montréal, Quebec Canada

**Keywords:** Parkinson’s disease, Limit of stability, Motor planning, Postural control, Computational modelling

## Abstract

Parkinson’s disease targets patients’ cognitive and motor abilities, including postural control. Many studies have been carried out to introduce mathematical models for a better understanding of postural control in such patients and the relation between the model parameters and the clinical assessments. So far, these studies have addressed this connection merely in static tests, such as quiet stance. The aim of this study is to develop a model for voluntary lean, and as such, identify the model parameters for both PD patients and healthy subjects from experimental data. The proposed model comprises planning and control sections. The model parameters for the planning section were extracted from the time response characteristics. Parameters for the control section were identified based on the spatial characteristics of the center-of-pressure (COP) response using an optimization process. 24 PD patients along with 24 matched healthy subjects participated in the study. The results showed a significant difference between the two groups in terms of temporal parameters for the planning section. This difference emphasizes bradykinesia as an essential symptom of PD. Also, differences were found for the postural control section. In all directions, the proportional gain of the feedback controller was significantly larger in PD patients; however, the gain of the feedforward controller was significantly smaller in PD patients. Furthermore, the control gains were strongly correlated with the clinical scales (Functional Reach Test and Unified Parkinson's Disease Rating Scale) in certain directions. In conclusion, the new model helps to better understand and quantify some PD symptoms in voluntary lean tasks.

## Introduction

Parkinson's disease (PD) is the second most common neurological disease after Alzheimer's. It negatively impacts both motor and cognitive performances. In order to better understand and classify the corresponding motor deficiencies, researchers have identified and proposed models and quantitative measures. These measures are supposedly more responsive than subjective qualitative assessments and therefore more appropriate for capturing minor improvements [1, 2]. Computational biomechanical models of postural control have been among the new approaches for a better understanding of how the disease affects motor performance.

Postural control models provide a unified and systematic framework for understanding and analyzing clinical and experimental tests related to balance and stability. These models allow for the representation and explanation of clinical tests through common descriptive terms, akin to stability criteria from control engineering [3, 4]. This approach enables researchers to create biomechanical models with high-level controllers, and the dynamic adjustment of controller parameters during simulation offers insights into the neuromechanical system governing human posture, with each parameter having a consistent and intuitive interpretation across task types [5]. During the last decades, various models have been proposed for postural control. In some studies, intermittent control strategy has been applied for studying human postural control [6–8]. The hypothesis is that active control is not a continuous function [6, 9]. Rather, it is activated intermittently. For example, in [6], an intermittent control is used for investigating the possible mechanisms of instability using sway data from PD patients as well as elderly and healthy young individuals. In their model, the active controller is state-dependent, meaning that its activation is decided based on the phase plane regions (angular position vs. velocity of the body). Their results show two groups of people with different postural control strategies; one group, mostly healthy individuals, use intermittent control strategy and the other group, mainly PD patients and elderly individuals, use continuous postural control strategy. Another study [9] compares two feedback controllers for stabilizing human quiet standing, considering the challenges of inadequate intrinsic ankle stiffness and a significant delay causing instability. The controllers examined are a standard linear continuous-time PD controller and an intermittent PD controller with a switching function defined in the phase plane. The intermittent controller, designed to switch off near the stable manifold of a saddle-like equilibrium and on otherwise, is found to be more robust than the standard model. The intermittent controller can utilize smaller feedback parameters, and its sway patterns involve a smart combination of slow motion along the stable manifold and spiral motion with low feedback gains, achieving overall dynamic stability. The study introduces a dead zone in the intermittent controller to enhance similarity with biological sway patterns without altering stability properties. In the frequency domain, the intermittent controller exhibits power spectral density functions characteristic of physiological sway movements in humans, contrasting with the standard model's over-damped second-order system characteristics.

Continuous postural control, on the other hand, is another control strategy that is studied for various tests. For example, Peterka [10] developed a feedback control model for healthy subjects, assuming an inverted pendulum around the ankle joint, for static tests, and generated center-of-pressure (COP) data for a set of control parameters through model simulations. By extracting Stabilogram Diffusion Function (SDF) parameters through a wide range of control parameters (PID controller gains), they concluded that there are correlations between SDF parameters and control model parameters. In their later study, Maurer et al. [11] investigated the potency of the proposed model for the examination of motor abnormalities in PD as compared to healthy subjects, as well as the effects of different medical and surgical therapies in PD. Using the results of this study, Rahmati et al. [12] studied the effects of rehabilitation exercises on the balance performance of PD patients. Using a trained Artificial Neural Network, they identified the control parameters for PDs before and after a rehabilitation training program and healthy subjects. Their results showed significant differences between the two groups and between PDs before and after rehabilitation. They also used postural control models for the frequency analysis of subjects’ COP responses to evaluate the covert extent of stability in PD, in static tests [5]. They found different gain and phase margins in PD patients in comparison with healthy subjects using postural control models in the frequency domain. Although most studies have used an inverted pendulum model, Kim et al. [13] developed a full-state feedback controller with a two-segment body dynamic model to simulate hip and ankle joints kinematics and kinetics in response to perturbations. Their results revealed differences between ankle and hip control gains of PDs compared to healthy subjects. Finally, Rahmati et al. [14] used a feedback postural control model in a clinical application, examining flexibility and stability degrees in PD patients based on the identified control gains. Based on their analysis, ${K}_{n}$, the noise gain, and ${K}_{p}$, the proportional gain of the controller, quantify the flexibility and stability degrees, respectively.

To the best of our knowledge, the mathematical modelling of the postural control of PD patients has been limited to static performances. Dynamic tests, on the other hand, may provide a more in-depth manifestation of the underlying ruling mechanisms which shape the overall postural control performance. Such tests involve subjects' voluntary movements, such as the Limit of Stability (LoS) test, in which subjects voluntarily lean around the ankle joint in different directions trying to move their COP towards certain target points on the ground. Dynamic tests are of high importance in understanding the balance and posture control in PD patients. These tests require the coordination of different control systems (e.g., internal modelling and sensory feedback) in the human brain [15, 16]. The LoS test, as a kind of voluntary dynamic test, in particular, challenges CNS motor planning, which can be performed in various directions and therefore reveal the direction dependency of the underlying postural disorders.

As a natural extension to the pertinent literature, this study intends to further develop the postural control models to dynamic reaching, particularly the LoS test as explained above, and identify the parameters of the control model for both PD patients and healthy subjects. The model and its parameters were investigated to elaborate on the fundamental characteristics and abnormalities of the dynamic postural control performance in PD patients.

## Materials and methods

### Dynamic reaching test and the experimental setup

**Fig. 1:**
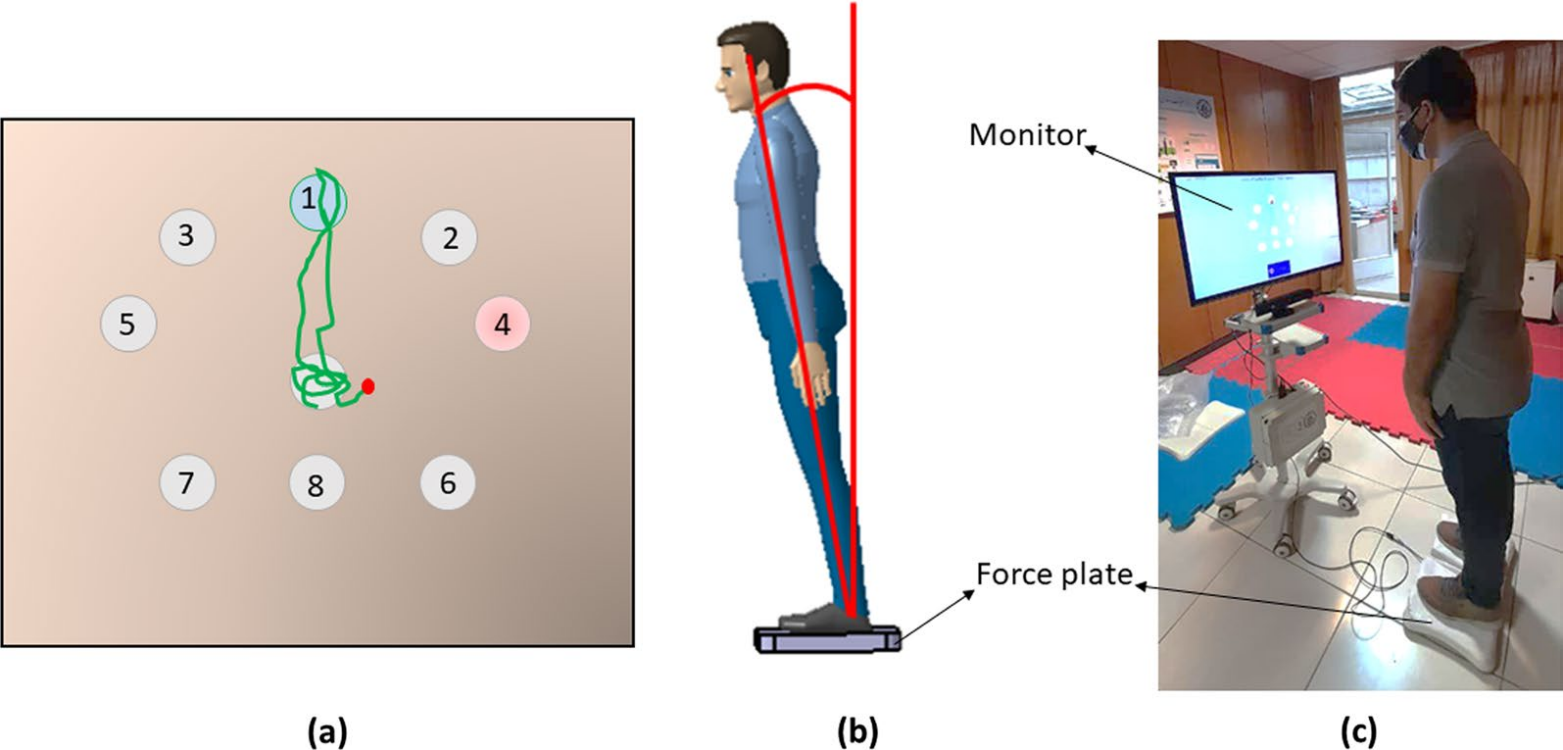
A sample of dynamic reaching test (LoS). **a** The active target is in pink, which is supposed to be hit by the COP (the small red circle) by voluntary lean. When the target is hit, the color changes to blue. The COP trajectory is in green. **b** The corresponding posture movement towards the target (the subject has to lean toward the target using ankle strategy); **c** The experimental setup

In the LoS test, as a dynamic reaching test used in this study, eight targets are located at different angles with combinations of A–P (anterior–posterior) and M–L (medio–lateral) directions. The subject observes the targets and his COP in real-time as small circles on a display monitor. When a target is illuminated, the subject leans toward it, moving his COP to reach and hit the target, and then returns to the upright stance (Fig. 1).

The experimental setup mainly consists of a force plate, with the sampling frequency of 80 Hz, recording the participant's COP and a monitor for displaying the targets and the COP position (Fig. 1). The force plate used in this study was custom made and had a fair concurrent validity (ICC = 0.818–0.989) compared with Kistler force plate as the reference (Kistler group, Switzerland) [17]. Also, the positions of all targets, with respect to the central point, are determined at the beginning of the test. The targets are located at 30% of the subjects' theoretical limits of stability found from Eq. (1):

1 $$d=0.3\times {H}_{CoM}\times \mathit{tan}\left(\theta \right),$$

where *d* is the distance of the target from the central point, ${H}_{COM}$ is the height of the subject’s center of mass, and $\theta$ is the theoretical limit of the leaning angle in each different directions. In the AP direction, the values for $\theta$ are 6.25° (target 1 (Fig. 1a), 4.5° (targets 2 and 3), 1.7° (targets 4 and 5), and 4.4° (targets 7, 8, and 9). In the ML direction, these values are 0° (targets 1 and 8), 6° (targets 2, 3, 6, and 7), and 8° (targets 4 and 5) [18, 19].

The COP trajectories of the subjects while performing the tests were recorded. Before the commencement of the data collection, the procedure was explained to each participant. Then, they did the test once to familiarize themselves with the setup (no data was collected). Next, they performed and repeated the tests four times. At the beginning of the test, the subjects had to stay in the upright stance position for 5 s. This was used to find their baseline upright posture (central position of the COP) and calibrate the positions of the targets accordingly.

The clinical assessments of the PD patients included Unified Parkinson's Disease Rating Scale (UPDRS) [20] and Functional Reach Test (FRT) [21] and were performed by a clinician. The clinical results were used to compare and validate the control parameters.

**Fig. 2:**
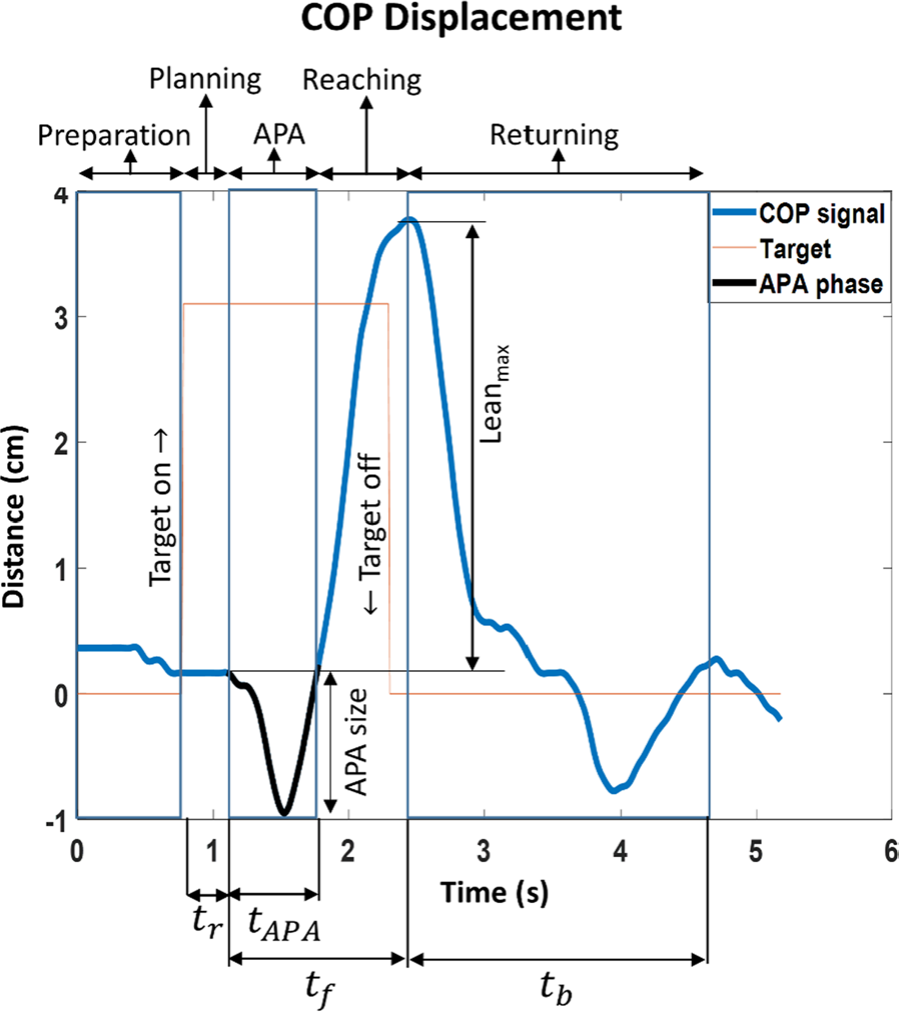
COP displacement and its segments. Preparation: the time before the target turns on; Planning: the time required for the CNS to plan for the movement; APA: a COP movement for counteracting the expected mechanical effects of the perturbations due to the reaching movement; Reaching: from the end of the APA until reaching the targets; Returning: from the end of the reaching until returning to the initial level. ${t}_{r}, {t}_{APA}, {t}_{f},\mathrm{ and} {t}_{b}$ are reaction time, APA time, reaching time, and returning time, respectively, ‘APA size’ is the maximum displacement of the COP during the APA, and ‘Lean_max_’ is the distance between the COP signal in the upright position and its maximum value after hitting the target

A sample of the COP displacement during a test is shown in Fig. 2. The displacement signal is divided into five sections: (1) Preparation, (2) Planning, (3) Anticipatory Postural Adjustments (APA), (4) Reaching, and (5) Returning [22, 23]. ‘Preparation’ refers to the duration before the target turns on. The subject is expecting the target, but there is no action. When a target turns on, the CNS requires a time, known as reaction time (${t}_{r}$), to initiate internal planning. Before moving toward the target, anticipatory postural adjustment (APA) occurs, which is an undershoot in the opposite direction of the target position. APA is commonly observed in voluntary movements. The CNS considers the act of reaching as a perturbation to the existing upright posture and counteracts this perturbation before the reaching movement starts by performing the APA [24]. Also, since APA is not based on any sensory information, the CNS controls the APA phase using an internal model [24]. As recommended in [22], a threshold-based algorithm was used to detect the onset of APA, with the threshold set at twice the standard deviation of the initial signal.

Reaction time, ${t}_{r}$, was considered as the time between the onset of the target illumination and the start of the APA. The end of the APA was determined as the time the COP returned to the baseline [22]. Thus, the APA duration (${t}_{APA}$) is the time between the onset and the end of APA, and the APA size is the peak-to-peak value of the COP displacement in the APA phase (Fig. 2). As shown in the figure, Reaching time (${t}_{f}$) is from when the APA starts until the COP displacement reaches the maximum value. Finally, Returning time (${t}_{b}$) is the duration of the COP returning back from its maximum peak to the next small peak that emerges after the COP is settled around (or passed) its initial value.

### The proposed model for dynamic reaching

**Fig. 3:**
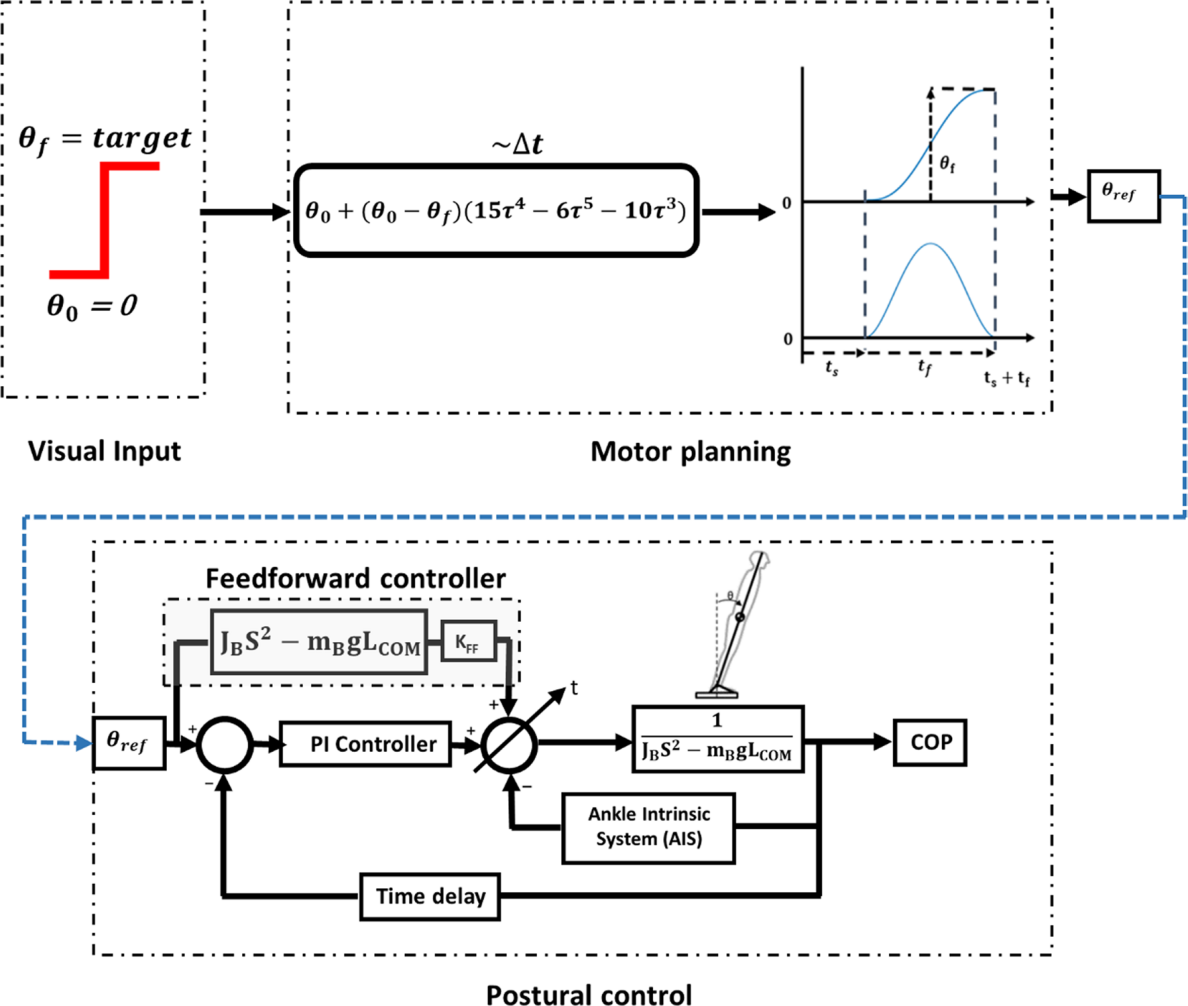
The proposed model for the dynamic reaching test. 'Visual input' is a step function which indicates the target appearance; 'Motor planning' is the trajectory planning section of the task; 'Postural control' is a feedback/feedforward control model for performing the motion commanded as $\theta _{{ref}}$ from motor planning module, completing the whole task of reach (hitting the target).”

The proposed model is shown in Fig. 3. The model consists of two main sections; ‘motor planning,’ which mimics the CNS in planning the task, and the ‘postural control’ section, which models the neuromechanics of the motion assuming only the usage of ankle strategy. After receiving the visual input as a step function (i.e., a target turns on), the CNS performs motion planning (motor planning section); according to the pertinent literature [25–27], the CNS constructs and uses an internal model for optimal planning of the reaching task, which typically has a bell-shaped velocity profile; the created path is the input of the second section of the model, i.e. the postural control section [28]. In the postural control section, the task is performed based on the CNS commands and visual feedback. The feedforward controller was added to account for producing the anticipatory postural adjustment (APA) [29, 30].

#### Motor planning

To the best of our knowledge, there is no investigation on motion planning in lower-limb dynamic reaching tests. In upper extremities reaching tests, however, there are several works which proposed a bell-shaped velocity profile based on the motion optimality hypothesis [31–33]. These studies used a polynomial expression as a model for the movement trajectory [25, 31, 34]. A similar formulation was used here for the body lean angle (Eq. 2):

2 $${\theta }_{ref}={\theta }_{0}+\left({\theta }_{0}-{\theta }_{f}\right)\left({A}_{1}{\tau }^{4}-{A}_{2}{\tau }^{5}-{A}_{3}{\tau }^{3}\right), \tau =\frac{t-{t}_{r}}{{t}_{f}},$$

where ${\theta }_{ref}$ is the reference angle of the body to move toward the target, ${\theta }_{0}$ and ${\theta }_{f}$ are the angle of the body at the initial and target points, respectively; ${t}_{r}$ and ${t}_{f}$ are reaction time and reaching time, respectively; ${A}_{1}, {A}_{2}, {\text{and}} {A}_{3}$ are constants that determine the geometry of the bell-shaped pattern taken from [25].

#### Postural control

The postural control section, as shown in Fig. 3, is an extension of the models previously proposed for static tests [12, 14]. It consists of two major control paths, feedforward (FF) and a time-dependent summation block of feedback (FB) controllers.

Most of the studies on APA found that the CNS uses a feedforward controller for this phase of the movement [24, 30, 35]. Even the APA in the gait initiation is shown to be a feedforward control [29]. Therefore, the feedforward controller, in the proposed model, is considered for the APA section, and the remaining is feedback controlled. It is assumed that the CNS shifts smoothly from a feedforward to feedback control as the reaching task progresses. As a result, a time-dependent transition is proposed in this study. As shown in Fig. 3, a summation block, in which the weights of the three control signals are regulated through coefficient $\alpha$, is added to the control model and defined as follows (Eq. 3):

$$\alpha =\frac{t-{t}_{r}}{{t}_{APA}}$$

3 $$\left\{ {\begin{array}{*{20}c} {t_{r} < t < t_{r} + t_{{APA}} } & {\tau = \tau _{{FF}} \times \alpha + \tau _{{FB}} \left( {1 - \alpha } \right) + \tau _{{AIS}} } \\ {t > t_{r} + t_{{APA}} } & {\tau = \tau _{{FB}} + \tau _{{AIS}} } \\ \end{array} } \right.,$$

where, $t$, ${t}_{r}$, and ${t}_{APA}$ are time, reaction time, and the duration of the APA phase, respectively. Also ${\tau }_{FB}$,${\tau }_{FF}$, and ${\tau }_{AIS}$ are the output torques provided by feedback, feedforward, and ankle intrinsic biomechanics, respectively. The latter refers to the intrinsic torque provided by the stiffness and damping of the ankle joint, and their values are taken from the literature [12]. $\tau$ is the total torque; $\alpha$ is a time-varying parameter from 0 (at the beginning of the APA) to 1 (at the end of APA). The feedback controller is a *PI* controller (Eq. 4):

4 $${\tau }_{FB}={K}_{P}\left({\theta }_{ref}-\theta \right)+{K}_{I}\left({\dot{\theta }}_{ref}-\dot{\theta }\right),$$

with ${\theta }_{ref}$ is from Eq. (2), *K*_I_ $=5 N.m/deg/s$ [12, 14], and ${K}_{P}$ was determined subject-specifically. The feedforward controller is an inverse model of the system (inverted pendulum) with ${K}_{FF}$ gain to be determined for each subject (Eq. 5):

5 $${\tau }_{FF}={K}_{FF}\left({J}_{B}{s}^{2}-mg{L}_{COM}\right),$$

where ${J}_{B}$, *m*, and ${L}_{COM}$ are moment of inertia, mass, and height of COM of the subject, respectively, and $g=9.81\frac{m}{{s}^{2}}$ is the gravitational acceleration.

There are two unknown parameters, ${K}_{P}$ (proportional gain in the feedback controller) and ${K}_{FF}$ (feedforward gain), in the postural control section that were identified subject-specifically. These parameters were identified based on two COP parameters, Lean_max_ and APA size, extracted from the experimental data (Fig. 2). In other words, ${K}_{P} \, \text{and} \, {K}_{FF}$ values were identified such that the error between Lean_max_ and APA size of the model and experiment were minimized at the same time (Eq. 6).

6 $${\text{minimize}}\left\{\begin{array}{c}\left|Lea{n}_{{\text{max}}(model)}-Lea{n}_{{\text{max}}(experiment)}\right|\\ \left|APA siz{e}_{model}-APA siz{e}_{experiment}\right|\end{array}\right..$$

**Fig. 4:**
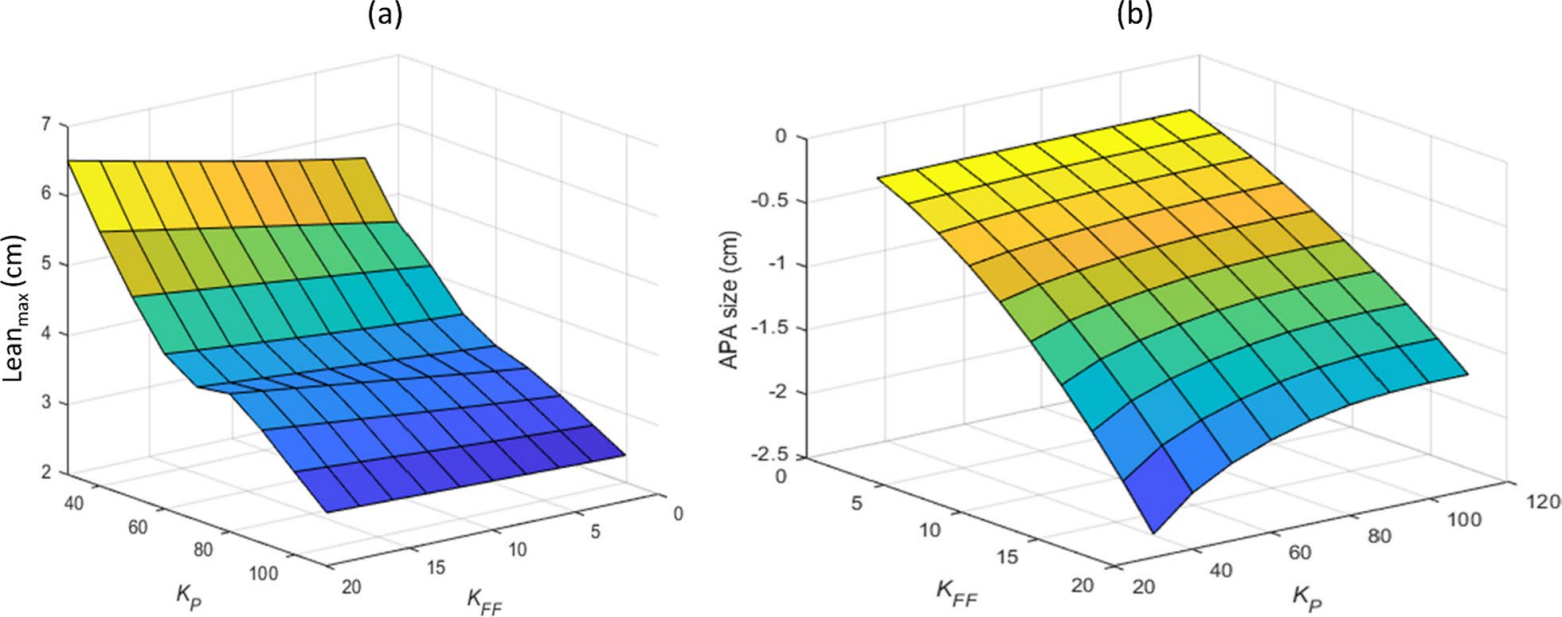
The effects of *K*_*FF*_ and *K*_*P*_ on the COP. **a** Lean_max_, **b** APA size

Also, as it is shown in Fig. 4, the variation of APA size and Lean_max_ values are smooth and there is no concern about local minima.

### Participants

Twenty-four PD patients ($61.5\pm 9.6 {\text{yrs}})$, with level of 1 to 3 according to the Hoehn and Yahr scale ($2.37 \pm 0.74)$ and disease duration of $8.9 \pm 5.4 {\text{yrs}}$, and 24 healthy subjects ($54.9\pm 7.4 {\text{yrs}})$ participated in this study (Table 1). The data for PD patients, diagnosed by a neurologist, was taken from a previous study in the same research group [18]. Healthy subjects had no previous balance disorders. Clinical assessments (UPDRS and FRT in Table 1) were done for PD patients to assess their balance function. The test protocol was approved by the ethics committee of the Iran University of Medical Science (IR.IUMS.REC.1400.826). All participants provided written confirmed consent according to the Declaration of Helsinki.

**Table 1:** Demoghraphy of the participants

Parameter (unit)	Healthy: mean $\pm {\text{SD}}$	Patients: mean $\pm {\text{SD}}$
Demographic data		
Age (year)	$54.9 \pm 7.4$	$61.5 \pm 9.6$
Gender	$24\mathrm{ male}$	$9\mathrm{ female}, 15\mathrm{ male}$
Height (cm)	$175.6 \pm 8.8$	$164.5 \pm 8.4$
Weight (kg)	$77.28 \pm 12.7$	$68.5 \pm 11.5$
Clinical measures		
MMSE^a^	–	$26.6 \pm 3.7$
HADS^b^	–	$5.12 \pm 3.1$
BDI^c^	–	$13.75 \pm 9.8$
UPDRS^d^	–	$26.14\pm 4.4$
FRT^e^	–	$31.5\pm 10.2$

^a^The Mini-Mental State Examination [36]

^b^Hospital Anxiety and Depression Scale [37]

^c^Beck Depression Inventory [38]

^d^Unified Parkinson's Disease Rating Scale [20]

^e^Functional Reach test [21]

### Data analysis

COP data collected from each subject was used to identify a subject-specific set of model parameters. For this purpose, each trial was first divided into eight sections (i.e. directions of targets). Then, Reaction time (${t}_{r}$), Reaching time (${t}_{f}$), Return time (${t}_{b}$), Lean_max_ and APA size (Fig. 2), and APA duration (${t}_{APA}$) were extracted for each direction. The averages of these parameters for each direction over four trials were used to identify the model parameters. Reaction time, Reaching time, and Return time were used to identify the motor planning parameters. Lean_max_ and APA size were used for the identification of the control gains (${K}_{P}$ and ${K}_{FF}$) as explained in "The proposed model for dynamic reaching". Finally, the independent t-test was used to compare the control parameters in each direction between the groups of healthy subjects and PD patients. The significance level was set to 0.05.

## Results

### Motor planning and temporal parameters

**Fig. 5:**
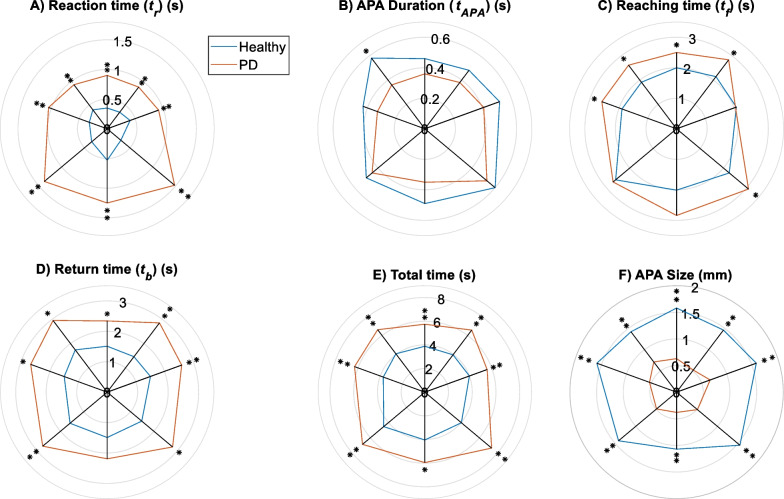
Temporal parameters (**A**–**E**) and APA size (**F**) for PD patients and healthy subjects in eight directions; *$p$ < 0.05,**$p$< 0.01

The temporal parameters identified for the subjects of the two groups are shown in Fig. 5A–E and Table 2. All parameters, except the APA duration, show significant differences between PD and healthy groups in most directions. APA duration (Fig. 4B) does not indicate a difference between the two groups (except in the left-forward direction). In order to create the motor planning section of the proposed postural control model, ${t}_{r}$ (reaction time) and ${t}_{f}$ (reaching time) as shown in Fig. 2 were used.

**Table 2:** Temporal parameters: reaction time (${t}_{r}$), APA duration (${t}_{APA}$), Reaching time (${t}_{f}$), Return time (${t}_{b}$), and Total time (${t}_{total}$), as well as APA and Lean_max_ size for both healthy individuals and PD patients

Parameter	Group	Target (Fig. 1a)							
		T1	T2	T3	T4	T5	T6	T7	T8
${t}_{r}$(s)	PD	0.9	0.88	0.93	0.92	1.05	1.48	1.38	1.25
	H	0.35	0.35	0.4	0.41	0.32	0.3	0.34	0.53
${t}_{APA}$(s)	PD	0.36	0.38	0.36	0.41	0.33	0.53	0.45	0.35
	H	0.46	0.48	0.58	0.52	0.43	0.60	0.50	0.49
${t}_{f}$(s)	PD	2.50	2.83	2.61	2.05	2.60	3.07	2.71	2.84
	H	2.00	2.14	1.91	2.08	1.90	2.25	2.60	2.01
${t}_{b}$(s)	PD	2.34	2.85	2.95	2.6	2.67	2.80	2.76	2.19
	H	1.51	1.46	1.74	1.51	1.50	1.48	1.59	1.49
${t}_{total}$(s)	PD	5.74	6.57	6.61	5.59	6.31	7.35	6.86	5.95
	H	3.88	3.95	4.06	4.00	3.73	4.02	4.53	4.04
APA size (cm)	PD	0.63	0.52	0.71	0.67	0.54	0.51	0.49	0.38
	H	1.58	1.46	1.42	1.59	1.59	1.55	1.42	1.07
Lean_max_ size (cm)	PD	4.32	4.49	4.51	4.69	4.46	4.11	3.988	2.60
	H	5.00	6.14	6.46	6.73	6.68	6.25	6.34	4.25

### Control gains

**Fig. 6:**
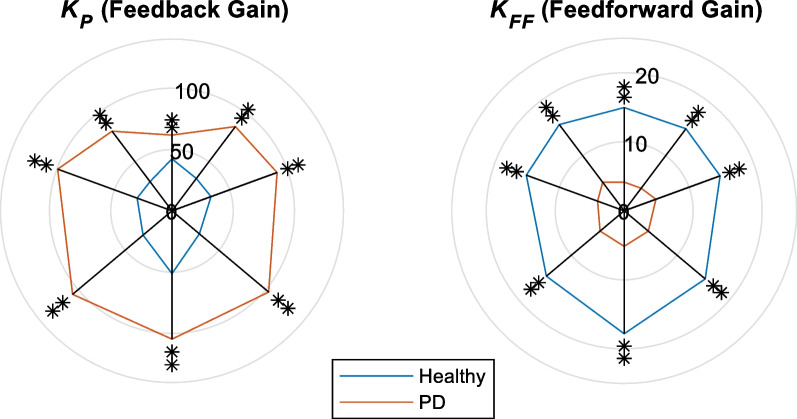
*K*_P_ and *K*_FF_ for PD patients and healthy subjects; *$p$<0.05, **$p$<0.01

The results for the control gains are shown in Fig. 6 and Table 3. In all directions, there are significant differences ($p<0.01$) between the two groups. ${K}_{P}$ (the proportional gain of the feedback controller) for PD patients is generally higher than that of the healthy subjects, while there are some differences between directions. The ${K}_{P}$ seems to decrease as the leaning direction shifts from the most backward to the most forward. Also, ${K}_{FF}$ (feedforward controller gain) in healthy subjects is generally higher than in PD patients.

**Table 3:** Control parameters ${K}_{P}$ and ${K}_{FF}$ for both healthy individuals and PD patients

Parameter	Group	Target (Fig. 1a)							
		T1	T2	T3	T4	T5	T6	T7	T8
${K}_{P}$	PD	61.73	86.20	81.27	91.30	99.32	103.01	105.93	104.60
	H	42.40	33.08	29.80	33.90	30.30	29.22	30.81	51.20
${K}_{FF}$	PD	4.16	4.09	5.18	4.90	4.12	4.57	4.56	5.14
	H	14.95	14.88	15.64	14.80	15.10	15.34	14.72	17.8

**Fig. 7:**
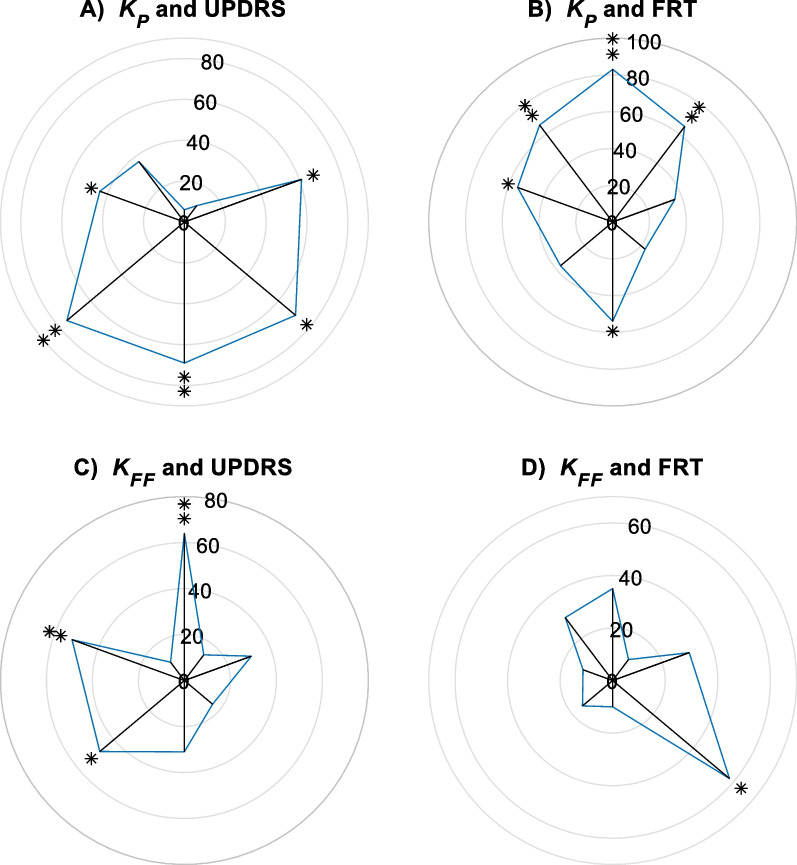
Correlations of ${K}_{P}$ and ${K}_{FF}$ with clinical measures, *$p$<0.05, **$p$<0.01. Correlations between ${K}_{P}$ and FRT are negative

Figure 7 shows the correlation (Spearman’s rank correlation test) between the control gains and the clinical measures. It is shown that *K*_P_ has significant, positive and negative correlations with Unified Parkinson’s Disease Rating Scale UPDRS) and Functional Reach Test (FRT) measures, respectively. ${K}_{P}$, in backward directions, is mainly correlated with UPDRS score, while ${K}_{P}$ in forward directions is mostly correlated with FRT. ${K}_{FF}$ has significant correlations with only three directions (1, 5, and 7 according to Fig. 1a). Also, its correlation with the FRT score is significant only in one direction (not shown in the figure).

## Discussion

In this study, a neuromechanical model for the dynamic reaching task in the form of voluntary lean, was proposed and used for a better understanding of the posture control in healthy and PD patients. The model consists of two sections: motor planning and postural control. Experimental data collected from the participants were used to identify the subject-specific parameters of the model. Statistical analysis showed significant differences between the model parameters in patients and healthy subjects. The following sections present and discuss possible explanations for these differences to better understand PD patients' behavior in dynamic reaching tasks.

### Temporal parameters

Bradykinesia, or slowness, is one of the most pivotal known motor symptoms in PD patients [39, 40]. Compared to the healthy group, larger time parameters, except for APA duration, suggest this symptom in PD patients. Larger time parameters were observed in other studies as well [22, 41]. The APA size and bradykinesia in PD patients may help to understand the reason for no significant difference between the two groups in terms of APA duration. As it is shown in Fig. 4F, the APA size in all directions for healthy subjects are significantly larger than those of PD patients, showing that healthy subjects travel a longer path through their APA compared to PDs. Nevertheless, healthy subjects exhibit faster movements when compared to PD patients, which effectively offsets the impact of the longer path. In other words, even though PD patients have slower movements, due to their shorter APA, they ultimately exhibit a similar APA duration when compared to healthy subjects. Moreover, there are some contradictory results in the literature, showing both longer [42] and no change of APA duration [22]. It seems that PD stage may be a major factor [43]. For example, PD patients in those studies reporting no significant change of APA duration [22], including this study, have lower averages of Hoehn and Yahr levels (2.39 in this study and 1.9 in [22]) than those reporting larger APA duration (2.72 in [42]).

### Feedback control gain (${{{K}}}_{{{P}}}$)

This parameter, as known from the classical control theory, adjusts the stiffness of the ankle joint in the model. As seen in Fig. 6, ${K}_{P}$ in PD patients is higher than in healthy subjects (*p* < 0.01 in all directions), which is in contrast with the results previously obtained in static tests [12, 14]. Similarly, when examining the anthropometric data presented in Table 1 for both PD patients and healthy individuals, it becomes evident that the proportional gain achieved in the quiet stance test with intermittent control, as reported in [6], shows a discrepancy as well. Rather, intermittent control results align with the results from previous research that employed continuous postural control models during quiet stance [12]. This underscores the significance of the dynamic aspects of the test on neuromuscular system performance, which might be exhibited mostly through higher muscle co-contractions in LOS test in PD patients. Muscle co-contraction is reported in the literature [44–46] for PD patients, resulting in a more stiffened ankle joint. Such higher stiffness has reflected in a larger ${K}_{P}$ in the model through the identification process. Although there is no literature on the comparison of the co-contraction level between quiet stance and voluntary lean, our results indicate that the phenomenon is more pronounced in dynamic tests.

Furthermore, fear of falling, as another symptom of PD [47, 48], particularly in dynamic tests, intensifies the co-contractions resulting in higher ${K}_{P}$'s for PDs. Also, fear of falling in backward direction is more intense [49], resulting in larger difference in ${K}_{P}$ between healthy and PD subjects, which is confirmed by our results. As it is shown in Fig. 5, ${K}_{P}$ is higher in backward direction for both groups. Moreover, it appears that there is a pattern of decreasing ${K}_{P}$ values from the backward directions to the forward directions. In other words, the ${K}_{P}$ values tend to decrease as the direction of leaning moves from the most backward to the most forward direction.

Finally, as shown in Fig. 6, the correlations of ${K}_{P}$ with clinical measures (FRT and UPDRS) are direction dependent. The FRT measure is strongly (> 0.6) correlated with ${K}_{P}$ only in forward direction most probably because this test is based on a similar forward-reaching task. The UPDRS score, on the other hand, shows stronger correlations in the backward leaning task. UPDRS measure includes various motor assessments that reflect the severity of Parkinson's symptoms, such as rigidity. As discussed, this rigidity is reflected in ${K}_{P}$ values of the model. Also, as the literature shows [47, 48], PD patients have higher fear of falling and rigidity in backward directions, elucidating the higher correlations between UPDRS and ${K}_{P}$ in backward directions than forward ones.

### Feedforward control gain ($K_{FF}$)

${K}_{FF}$, found from the APA size, was seen to strongly distinguish between healthy and PD groups (p < 0.01), with much higher values in healthy subjects. This difference between the two groups can be justified through several observations. First, Initial Muscle Activation (IMA) is smaller in PDs than in the healthy group [50, 51]. Since the APA happens at the beginning of the movement, it suggests that PDs' weak IMA results in a smaller APA compared to the healthy group. Second, considering the definition of APA, the CNS predicts how a movement happens; therefore, CNS produces a movement in the inverse direction of the predicted one in order to adjust the whole movement and prevent instability. Production of reverse movement is based on CNS' prediction ability, supporting the possibility that the different APA size and ${K}_{FF}$ in healthy and PD groups stem from their different levels of prediction ability [52, 53]. Third, although the APA is introduced for adjusting the movement, it acts as a perturbation as well. It has been shown that PDs, compared to healthy subjects, have less control ability in static conditions when they are exposed to perturbations; likewise, since the reaching movement starts after the APA phase, subjects are still at their static condition during the APA phase [13]. Therefore, patients' CNS may adjust smaller APA in order not to perturb their condition more than their ability. Finally, in the APA phase, the CNS needs to coordinate and choose different controllers and strategies to perform a proper movement. This coordination and the processing burden for CNS during the APA phase may be another reason for having smaller APA in PDs. In other words, it seems that PD patients can better manage this coordination, linking the phases of movement and transition from one control strategy to another, when APA size is smaller with a lower processing burden.

As for the correlations between ${K}_{FF}$ and the clinical measures, previous studies claim that the APA size is weakly correlated with the clinical measures [22]. Our results on ${K}_{FF}$, if averaged over all 8 directions, supports the same observation. However as seen in Fig. 6C, the correlation pattern is highly direction dependent such that ${K}_{FF}$ has a strong correlation with UPDRS in forward direction. This reiterates the direction dependency of the dynamic reaching tests and its capacity to represent the disease severity.

It should be noted that this study has some limitations that should be considered when interpreting the results. First, healthy group is not gender-balanced, and this should be considered when comparing the results of this study with those of others. Second, all PD patients were in the level of 1–3 of Hoehn and Yahr criterion. Future studies should investigate the effects of disease with higher stages of Hoehn and Yahr on the postural control parameters.

## Conclusions

In this paper, a neuromechanical model was proposed for multi-direction voluntary lean or dynamic reaching tests to investigate the differences between Parkinson's disease (PD) patients and healthy subjects.

The study reveals significant differences in motor planning and postural control between PD patients and healthy subjects. The difference in the temporal parameters are believed to have roots in bradykinesia and slower movements in PD patients. The feedback control gain (${K}_{P}$) was higher in PD patients which is likely due to increased muscle co-contractions and fear of falling, especially in backward leaning. Additionally, the feedforward control gain (${K}_{FF}$) is lower in PD patients, indicating weaker initial muscle activation and CNS’s ability to predict possible disturbances from the voluntary movement. It was also revealed that the correlations between the control gains and the clinical measures are direction dependent. The feedback and forward control gains show stronger correlations with clinical measures in backward and forward leaning, respectively.

## Data Availability

The data analyzed during the current study are available from the corresponding author on reasonable requests.
